# Motor empathy is a consequence of misattribution of sensory information in observers

**DOI:** 10.3389/fnhum.2014.00047

**Published:** 2014-02-06

**Authors:** Indra T. Mahayana, Michael J. Banissy, Chiao-Yun Chen, Vincent Walsh, Chi-Hung Juan, Neil G. Muggleton

**Affiliations:** ^1^Institute of Cognitive Neuroscience, National Central UniversityJhongli, Taiwan; ^2^Department of Psychology, Goldsmiths, University of LondonLondon, UK; ^3^Department and Graduate Institute of Criminology, National Chung Cheng UniversityChiayi, Taiwan; ^4^Institute of Cognitive Neuroscience, University College LondonLondon, UK

**Keywords:** empathy, mirror mechanism, motor evoked potential, transcranial magnetic stimulation, peripersonal space, extrapersonal space

## Abstract

Human behavior depends crucially on the ability to interact with others and empathy has a critical role in enabling this to occur effectively. This can be an unconscious process and based on natural instinct and inner imitation (Montag et al., 2008) responding to observed and executed actions (Newman-Norlund et al., 2007). Motor empathy relating to painful stimuli is argued to occur via the mirror system in motor areas (Rizzolatti and Luppino, 2001). Here we investigated the effects of the location of emotional information on the responses of this system. Motor evoked potential (MEP) amplitudes from the right first dorsal interosseus (FDI) muscle in the hand elicited by single pulses of transcranial magnetic stimulation (TMS) delivered over the left motor cortex were measured while participants observed a video of a needle entering a hand over the FDI muscle, representing a painful experience for others. To maintain subjects’ internal representation across different viewing distances, we used the same size of hand stimuli both in peripersonal and extrapersonal space. We found a reduced MEP response, indicative of inhibition of the corticospinal system, only for stimuli presented in peripersonal space and not in extrapersonal space. This empathy response only occurring for near space stimuli suggests that it may be a consequence of misidentification of sensory information as being directly related to the observer. A follow up experiment confirmed that the effect was not a consequence of the size of the stimuli presented, in agreement with the importance of the near space/far space boundary for misattribution of body related information. This is consistent with the idea that empathy is, at least partially, a consequence of misattribution of perceptual information relating to another to the observer and that pain perception is modulated by the nature of perception of the pain.

## Introduction

Empathy has a significant role in the sharing of affective states and in predicting and understanding the feelings, motivations, and actions of others, and the showing of compassion (Gallese, 2003; Minio-Paluello et al., 2009; Bernhardt and Singer, 2012). It has been argued that for emotional social interactions, mirror neuron mechanisms may be involved in the neural basis of the observer’s empathy for the emotional state of another individual (Schulte-Ruther et al., 2007). It has been argued that, during observation of an action being executed, activation of mirror neurons matches the observed actions with internal representations (Gallese, 2003; Iacoboni and Mazziotta, 2007). Thus, this has been extrapolated to suggest that mirror neurons may provide a simulation-based form of empathy through interactions with the limbic system or other brain areas related to emotion (Iacoboni and Mazziotta, 2007). One example of reduced effectiveness of these mirror systems can be seen in autistic disorders such as Asperger syndrome (Caggiano et al., 2009) which is associated with reduced empathy and characterized by difficulties in social interaction as well as a narrowed range of personal interests (Minio-Paluello et al., 2009).

The subjective experience of pain may comprise autonomic activity and the desire to produce behavioral responses (Rainville, 2002), the so called pain empathy response. This response activates neural structures that are also involved in the direct experience of pain (Lamm et al., 2011). Observation of painful or non-noxious events shown on the body is said to result in functional modulation of the corticospinal system through the mirror neuron system (Avenanti et al., 2005) and lead to inhibition of corticospinal excitability. This can be observed by measurement of motor evoked potential (MEP) signals (Avenanti et al., 2009) and the MEP amplitude may be used to show the modulation of the motor system as a consequence of altered mirror system activity. Motor inhibition, as shown by a reduction in MEP amplitude specific to the muscle in which pain is observed, is found during the observation of needles penetrating body parts of a human model (Avenanti et al., 2006). Furthermore, tonic muscle pain in the hand may result in a long-lasting depression of the MEP amplitude resulting from transcranial magnetic stimulation (TMS) stimulation of the primary motor area in the hemisphere contralateral to the painful stimulation (Le Pera et al., 2001). This is therefore a good method for observation of changes, presumably modulation of corticospinal excitability, induced by pain and the mirror neuron system modulation of action. It is worth noting that similar stimuli have also been employed in conjunction with fMRI, showing responses in anterior cingulate cortex (Morrison et al., 2004).

Dynamic processes relating to peripersonal and extrapersonal space coding are important for perceiving the correct spatial position of target objects (Berti et al., 2002). Mirror functions in space have been investigated in monkey studies and those in the premotor cortex (F5) and anterior intrapariteal area (AIP) play a fundamental role in space and action perception relating to the spatial organization of movements (Rizzolatti and Matelli, 2003). These areas respond mainly to visual stimuli presented in peripersonal space (Graziano, 1997; Holmes and Spence, 2004) thus exhibiting spatial selectivity for subsequent types of behavioral responses. Examples of this include approaching behavior performed in extrapersonal space or competitive behavior in peripersonal space (Caggiano et al., 2009).

We hypothesized that different somatomotor responses might be observed in human when “mirror-matching” occurs when observing others’ feelings at different viewing distances. According to Avenanti et al. (2006), motor reaction to observation of pain that results in suppression of MEPs amplitude may be due to a *mirror-like resonance* mechanism that extracts basic sensory qualities of another person’s painful experience, for example: the location of the noxious stimulus. Our primary hypothesis was that such a change is potentially a consequence of misattribution of observed stimuli as relating to the body of the observer. Consequently, this leads to the prediction that effects of observed painful stimuli will be greater if they are presented in a position where it is more plausible that they are actual representations of the observer’s own body (i.e., in peripersonal or near space) than when they are in a position where this is less likely (i.e., extrapersonal or far space). We therefore manipulated the distances at which affective visual stimuli were presented to evaluate the effects on motor system excitability as an index of pain empathy responses.

## Materials and methods

### Participants

Eleven right handed subjects (5 males and 6 females, mean age: 24.2 ± 1.9 years) with no previous history of neurological problems, all with normal or corrected to normal vision, and without colorblindness participated in the experiment. Right handedness was determined using an adapted version of the Edinburgh Handedness Inventory. Prior to the experiment, participants were also required to verbally report any anxiety or phobia of needles or if they had any conditions involving prolonged use of drugs administered by injection (e.g., insulin-dependent diabetic mellitus). The presence of any of these would have resulted in exclusion from the study. All participants were naïve regarding the experiment task and gave informed consent prior to participation. This experiment was conducted in accordance with the Declaration of Helsinki and the protocol was approved by the local Ethics Committee.

### Electromyogram and transcranial magnetic stimulation (TMS) Recordings

A Magstim 200 Super-Rapid Stimulator was used to deliver stimulation via a 70 mm figure of eight coil (Magstim Co., Whitland, Dyfed, UK). The left motor cortex was located initially 5 cm left of the vertex and single pulses of TMS applied near this location to identify the best area to produce a twitch in the right first dorsal interosseus (FDI) muscle of the hand (the level of stimulation used depending on the responses in each subject). The minimum machine output intensity to produce a visually observed muscle twitch was identified using a modified binary search algorithm (Tyrrell and Owens, 1988; Thilo et al., 2004; Silvanto et al., 2007). The obtained intensity then was decreased to identify the resting motor threshold (rMT), rMT was defined as the minimum intensity to produce a peak to peak MEP of 50 μV in at least 5 out of 10 consecutive trials (or with 50% probability) in the relaxed FDI muscle (Rossini et al., 1994; Avenanti et al., 2005, 2006). TMS pulses during the experiment were delivered at an intensity of 120% of this resting motor threshold for each subject individually (mean intensity: 80.9 ± 14.2% of machine output). After the experiment session, none of the participants complained of or reported any discomfort related to the TMS received.

MEPs induced by single pulse TMS over the left motor cortex were recorded simultaneously from the right FDI and abductor digiti minimi (ADM) muscles during the experiment using the Biopac MP35 system (Biopac System, Inc, CA, USA) and were band-pass filtered (20 Hz–2.5 kHz), digitized (sampling rate 5 kHz) and stored for offline analysis to measure the mean peak-to-peak (p-p) amplitudes of twitches from the FDI and ADM muscles. The MEPs recorded from the ADM muscle location served as a control for the specificity of any changes seen in the FDI muscle activity during the experiment and reliable responses from this muscle were confirmed during the localization and thresholding of the FDI muscle.

### Procedure

Participants had to perform 8 blocks of trials, 4 blocks for each distance (near or far space) with 2 blocks of the pain and touch conditions. There were 24 trials per block and a TMS pulse was delivered every trial. Consequently there were 48 trials for each condition (pain or touch) at each distance. A trial started with a fixation cross for 1 s, followed by a video stimulus for 2.5 s, and followed by a blank screen for 7.5 s (similar to the long intertrial interval used by Avenanti et al., 2005). A single TMS pulse was delivered during the clip, when the needle had penetrated the hand (pain condition) or the cotton swab had touched (touch condition) the skin, both of which were over the location equivalent to the FDI muscle. MEPs elicited were collected. These stimuli have previously been used by Avenanti et al. (2005) and Minio-Paluello et al. (2009).

Participants were not given any information about the onset of TMS and instructed to watch carefully and pay attention to the video stimuli and asked to keep their right hand relaxed.

**Figure 1:**
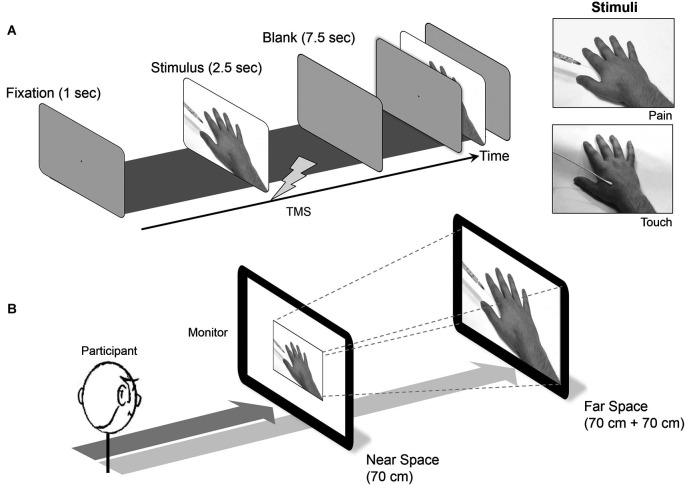
**(A)** The stimulation procedure. **(B)** The monitor distances: the peripersonal (near) space was fixed at 70 cm and the extrapersonal (far) space was at 140 cm distance, by adjusting a 19 inch-monitor position from the observer.

Presentation of the video stimuli was controlled with E-Prime (Psychology Software Tools Inc., Pittsburgh, PA) in color presentation and showed the same male right hand for all trials. The video stimuli were presented on a 19 inch cathode ray tube monitor, with 75 Hz refresh rate, either in near space or far space with the presentation order counterbalanced (see Figures 1A, B). The near space location was 70 cm from the observers and far space at 140 cm, fitting with the definition of near space as a distance within arm reach (Wooding and Allport, 1998; Weiss et al., 2000). The size of the video animations display were 15 × 10^˚^ of visual angle (size of the hand approx. 9.5 × 8.4^˚^ of visual angle) and were controlled in both the near and far conditions so there were no changes of the size (in terms of degrees of visual angle) of the hand pictured in the video. With this manipulation, in dim light experiment room, we expected that participants were unaware of the difference between two viewing distances.

Participants were seated comfortably either 70 or 140 cm away from the display with the center of the screen at eye level for both the near and far conditions. Head position was controlled by a chinrest. The right hand, with electromyography electrodes attached, rested on a table in front of the participant.

#### Follow up experiment

Following the experiment described above, a second, broadly similar experiment was conducted to evaluate whether any results obtained were affected by the size of the hand displayed in the far space condition (i.e., was the fact that it was essentially a large hand presented further away important). As such, the experiment was repeated as described above with the exception that the stimuli presented in near and far space were identical in size on this occasion (see Figure 3A). Twelve right-handed subjects (6 males and 6 females, mean age: 22.4 ± 2.3 years, mean TMS threshold 78.3 ± 7.7%) took part in this experiment.

### Data analysis

#### Subjective measures analysis

Subjective measures analysis was carried out to evaluate participants’ subjective perception of pain. In the subjective measures analysis, to assess participants’ perception of pain we used the short form McGill Pain Questionnaire (SF-MPQ), a multi-dimensional measure of perceived pain in adults, consisting of the Pain Rating Index (PRI), a visual analog scale (VAS), and Present Pain Intensity (PPI). All of the subjects were asked to rate the observed stimuli after the TMS session in order to minimize bias. The PRI was used to rate participants’ subjective pain perception and required them to imagine how the pain would feel if applied to them. This consists of 15 representative words that are rated on a 4-point *Likert*-type rating scale ranging from 0 (none) to 3 (severe) with 11 sensory and 4 affective words. Using a VAS (10-cm-long) and PPI (range from 0 to 5), participants were asked about the pain intensity shown in the video animation and whether participants considered the pain sensation represented in the video to be intense.

#### Motor evoked potential (MEP) analysis

The MEP data were recorded during the experiment for later analysis using Biopac BSL 4.0 software (Biopac System, Inc, CA, USA). The MEP data was processed offline and the trials with electromyogram (EMG) activity before TMS (less than 5% of trials) were excluded from analysis. The p-p MEPs amplitudes outside the mean ± 2 standard deviations were also excluded.

##### Correlation analysis of subjective measurements and motor evoked potential (MEP) amplitude change.

The indices of MEP amplitude change were computed as follows: amplitude during observation of the pain condition minus amplitude during observation of the touch hand condition divided by the average of the same two conditions. For the correlation of subjective measurements and MEPs amplitude change, *Pearson* correlation coefficients between indices of amplitude change of MEPs recorded from each muscle and subjective reports were computed in each experiment.

##### Motor evoked potential (MEP) amplitudes in near and far space.

Analysis of the MEP amplitudes was done with a within-subject repeated measures three-way analysis of variance (ANOVA) with distance (near and far), condition (pain and touch), and muscle (FDI and ADM) as within-subject factors. The MEP amplitudes recorded during “Needle in FDI” condition in near and far conditions were compared against the value of “Touch in FDI” condition in near and far conditions by means of paired-sample *t*-tests.

## Results

### The correlation of subjective measurements and Motor evoked potentials (MEPs) amplitude change

In the analysis of subjective measurements indexes, the mean of the sensory-PRI score was 19.2 ± 3.5 SD and the affective-PRI was 4.2 ± 3.0 SD. In each question, the sensory-PRI was higher than the affective-PRI (1.7 ± 0.3 vs. 1.1 ± 0.07 SD, *t*_(10)_ = 3.361, *p* = 0.007). Sensory-PRI analysis showed a predicted negative correlation with MEP amplitude change for the near viewing distance (*r* = −0.560, *p* = 0.037). For the far distance there was also a correlation but this was not significant (*r* = −0.502, *p* = 0.070). We found the video stimuli could induce perception of moderately intense pain (VAS: 4.9 ± 2.2 cm and PPI score 2.5 ± 1.4). Moderate scores of VAS and PPI indices showed that the observation of pain scene visual stimuli triggered emotional reactions of personal distress (Avenanti et al., 2009).

### Motor evoked potential (MEP) amplitudes

Analysis of the MEP amplitudes with a within-subject repeated measures three-way ANOVA with distance (near and far), condition (pain and touch), and muscle (FDI and ADM) as within-subject factors revealed a significant interaction (*F*_(1,10)_ = 10.742, *p* = 0.008). Two-way interactions of distance vs. muscle and condition vs. muscle showed no significant results (*F*_(1,10)_ = 1.121, *p* = 0.315 and *F*_(1,10)_ = 0.599, *p* = 0.457, respectively). A significant main effect of muscle was found (*F*_(1,10)_ = 6.580, *p* = 0.028), with no significant main effect of distance and condition (*F*_(1,10)_ = 0.540, *p* = 479, and *F*_(1,10)_ = 0.042, *p* = 0.841, respectively).

Separate two-way ANOVAs with factors of distance (near and far) and condition (pain and touch) were carried out for each muscle. In FDI muscle, a significant two-way interaction of distance vs. condition was found (*F*_(1,10)_ = 7.810, *p* = 0.019), with no significant main effect of distance (*F*_(1,10)_ = 1.617, *p* = 0.232) or condition (*F*_(1,10)_ = 0.279, *p* = 0.609). For the ADM muscle, no significant two-way interaction was found (*F*_(1,10)_ = 0.116, *p* = 0.740).

**Figure 2:**
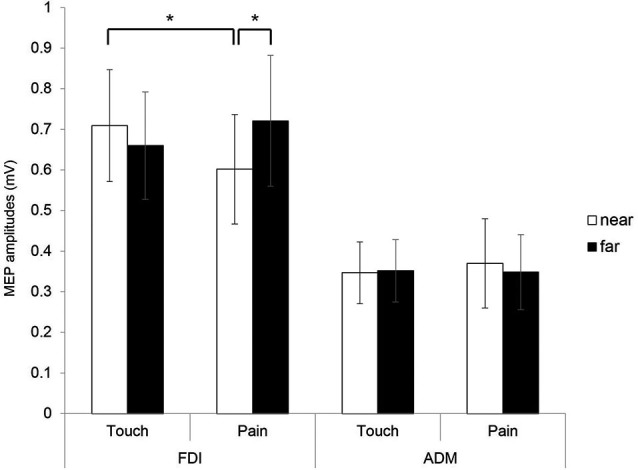
**MEPs amplitudes of the FDI and ADM hand muscles in near and far conditions (Error bars: Standard error means (SEM))**.

In *post-hoc* analyses, significantly lower FDI MEP amplitudes during the pain condition for the near distance were found when compared to amplitudes during the touch condition for the near distance (*t*_(10)_ = 2.73, *p* = 0.021) and amplitudes during pain condition for the far distance (*t*_(10)_ = −2.796, *p* = 0.019). This revealed that the display of actual painful stimuli delivered to the hand resulted in modulation of the motor cortex representing this area (potentially via an inhibition of corticospinal excitability) but only when presented in near space and not for far space (see Figure 2).

### Follow up experiment

**Figure 3:**
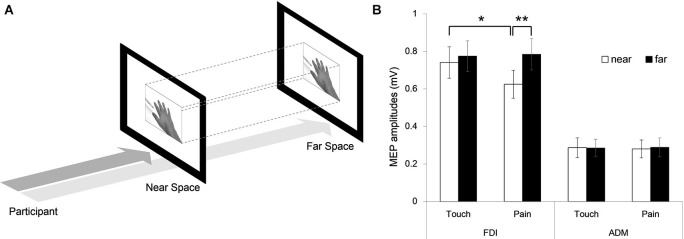
**Follow up experiment. (A)** Identically sized stimuli were presented in near and far space. **(B)** MEP amplitudes of the FDI and ADM muscles in the near and far viewing distance conditions (Error bars: SEM, * *p* < 0.05, ** *p* < 0.01).

Analysis was conducted in the same manner as the initial experiment. As before, a three-way ANOVA revealed a significant interaction (*F*_(1,11)_ = 5.471, *p* = 0.039). A significant two-way interaction of distance vs. muscle was found (*F*_(1,11)_ = 6.488, *p* = 0.027) with significant main effects of muscle and distance (*F*_(1,11)_ = 42.578, *p* < 0.001 and *F*_(1,11)_ = 6.447, *p* = 0.028, respectively). The main effect of distance may have been due to the differing visual angle of the stimuli in near and far space. Separate two-way ANOVAs were carried out for each muscle. In FDI muscle, a significant two-way interaction of distance vs. condition was found (*F*_(1,11)_ = 5.281, *p* = 0.042), with significant main effects of distance (*F*_(1,11)_ = 7.124, *p* = 0.022) and condition (*F*_(1,11)_ = 5.145, *p* = 0.044). In contrast, for the ADM muscle, no significant two-way interaction was found (*F*_(1,11)_ = 0.045, *p* = 0.836). In *post-hoc* analyses, the results were also similar to the initial experiment. In near space the FDI MEP amplitudes during the pain condition were lower compared to amplitudes during the touch condition (*t*_(11)_ = 2.800, *p* = 0.017) and also when compared with amplitudes during the pain condition for the far condition (*t*_(11)_ = −3.739, *p* = 0.003) (see Figure 3B). These results confirm that the initial findings of a lack of effect for the far pain condition were not a consequence of the size of the stimuli presented.

## Discussion

In this study, we investigated the pain empathy response for different viewing distances, looking at both near and far space. Results were consistent with previous studies that found a reduction in amplitudes of MEPs during the observation of needles penetrating body parts of a human model (Le Pera et al., 2001; Avenanti et al., 2005, 2006). Importantly, our study showed that the empathy response indexed by MEP modulation is limited only to peripersonal space. It was also in line with a study of spatial predictability of somatosensory targets by Van Damme and Legrain (2012) which suggested that spatial attention to a painful somatosensory stimulus is modulated only when the somatosensory targets were in near locations. In the present study, the reduced MEP seen only for near space pain related stimuli suggests is consistent with it being a consequence of misidentification of sensory information, with the MEPs being unaffected by far space stimuli. This effect was also found regardless of whether the stimuli were presented with similar retinal sizes or in smaller with greater distance.

When the painful stimulus is near, it may activate the detection system to facilitate the processing of behaviorally significant sensory input and to select the appropriate response (Legrain et al., 2011). As a painful sensation is unsurprisingly identified as something to be avoided, it is particularly important to monitor nearby objects in order to coordinate avoidance and defense with the aim of preventing potential physical threats, maintain the physical integrity of the body and avoid tissue damage (Cooke and Graziano, 2004; Van Damme and Legrain, 2012).

Empathy is the ability to appreciate the emotions and feelings of others with a minimal distinction between the two (Decety, 2011).**** The use of painful video stimuli was expected to result in somatic resonance in pain processing areas for others and the self, and triggering empathic responses. The expression of pain also provides a crucial signal that can motivate comforting and caring behaviors in others. In peripersonal space, there is an emergent capacity for self-awareness that is linked to the development of more advanced forms of empathy and social attachment serves intrinsically important regulatory functions related to security, nurturing and distress alleviation (Decety and Svetlova, 2012). Furthermore, this function in peripersonal space is important in terms of human–human interactions for prosocial behavior such as shaking hands or kissing the cheek of another (Lloyd, 2009).

The empathy system related to motor excitability was modulated by stimuli in peripersonal space but seems to be unaffected when the stimuli were presented in extrapersonal space. An ability to disambiguate peripersonal from extrapersonal space allows the observer to evaluate interpersonal behaviors (Caggiano et al., 2009). Thus, it might be assumed that in extrapersonal space, the brain limits the ability to regulate emotions as brain function related to extrapersonal space is more important in producing action or in movement planning (Rosenbaum et al., 2001) rather than regulating responses that may relate to effects on the self.

Perception of an emotion or feeling in another individual activates neural mechanisms responsible for the generation of similar emotions (Gallese, 2003; Gallese et al., 2007). We show that the motor empathy response has a distance limitation. This suggests that empathy responses of this type may be, at least partially, a consequence of the misidentification of visual information as relating to the observer. This may explain (at least partially) findings such as the effects of race on empathy (Forgiarini et al., 2011) and also leads to the prediction that the empathy related modulation of the motor response should reflect the (perceived) similarity of the observer and the stimulus and be altered should the stimulus be presented in a manner which the observer would be unable to replicate (for example, using unusual hand positions).

## Conflict of interest statement

The authors declare that the research was conducted in the absence of any commercial or financial relationships that could be construed as a potential conflict of interest.
